# Patient trust and readiness for digital health-enabled precision care after heart transplantation: a real-world survey

**DOI:** 10.3389/fdgth.2026.1839950

**Published:** 2026-08-11

**Authors:** Chiara Tessari, Valentina Esenia, Marco Andreis, Andrea Bagno, Gino Gerosa

**Affiliations:** 1 Department Cardiac, Thoracic, Vascular Sciences and Public Health, University of Padua, Padua, Italy; 2 Department of Industrial Engineering, University of Padua, Padua, Italy

**Keywords:** digital health, digital literacy, heart transplantation, patient trust, precision medicine, privacy, remote monitoring

## Abstract

**Background:**

Digital health may support continuous, personalized follow-up after heart transplantation; however, successful implementation depends on patient trust, digital confidence, and acceptable data-governance arrangements.

**Objective:**

To characterize digital access, perceived usefulness, trust, privacy concerns, and implementation readiness among adult heart transplant recipients, and to explore differences across demographic and technology-use subgroups.

**Methods:**

In this cross-sectional study, consecutive adult heart transplant recipients attending routine follow-up at a tertiary center completed a 43-item questionnaire developed through literature-informed item generation, multidisciplinary expert review, and pilot testing in 5 transplant recipients. Responses were summarized descriptively. Exploratory subgroup comparisons used Pearson chi-square or Fisher exact tests, as appropriate. As multiple comparisons were exploratory, *p*-values were interpreted as hypothesis-generating.

**Results:**

Ninety-three recipients participated; 61 (65.6%) were male and 74 (79.6%) were older than 45 years. Smartphone use was common (85/93, 91.4%), whereas wearable use remained limited (18/93, 19.4%). Most respondents agreed that technology could improve health monitoring (69/92, 75.0%), and 69/93 (74.2%) considered digital tools more useful and reliable when incorporating guidance from their treating clinicians. Nevertheless, 29/93 (31.2%) expressed discomfort with device-led monitoring, and 49/92 (53.3%) preferred physician advice over app-based guidance. Concerns about data misuse were reported by 44/93 (47.3%), although 72/93 (77.4%) supported data use for clinical research and 69/93 (74.2%) valued revocable consent. Perceived usefulness differed significantly by sex (males 83.3% vs. females 59.4%, *p* = 0.023). Medication-related insecurity and preference for physician advice varied across age groups (*p* = 0.029 and *p* = 0.030, respectively). Greater daily technology use was associated with less medication-related insecurity and less discomfort with monitoring (*p* = 0.009 and *p* = 0.022).

**Conclusions:**

Heart transplant recipients demonstrate substantial but conditional readiness for digital health adoption. Successful implementation appears to depend not only on access, but also on clinician integration, task-specific confidence, and transparent, patient-controlled data governance. These findings support a staged, human-centered implementation approach rather than technology substitution for established clinical relationships.

## 1 Introduction

Heart transplantation is a life-saving treatment for end-stage heart failure; however, its benefits depend on lifelong surveillance, adherence to complex therapeutic regimens, and the timely recognition of rejection, infection, graft dysfunction, and other complications. Conventional follow-up is largely organized around scheduled outpatient encounters. Although indispensable, such episodic visits provide only intermittent information and may fail to capture clinically meaningful changes occurring between appointments.

Mobile applications, wearable sensors, patient portals, and remote monitoring platforms offer the potential for longitudinal data capture and more responsive, individualized care. Across cardiovascular medicine, digital interventions have been shown to support symptom monitoring, rehabilitation, communication, and selected aspects of healthcare efficiency (1–5). In the transplantation setting, these tools could eventually integrate patient-generated data with clinical information to enable individualized surveillance and risk stratification (6). Their clinical value, however, remains contingent on sustained engagement, reliable data, equitable access, and seamless integration into professional workflows.

Implementation is therefore not a purely technical challenge. Digital literacy, confidence in using technology for high-stakes tasks, trust in clinicians and institutions, privacy expectations, and concerns that technology may replace rather than support human care all influence adoption. These issues may be particularly salient in heart transplant recipients, who are often older, clinically vulnerable, and highly dependent on an established relationship with a specialist team.

Evidence specifically addressing the perspectives of heart transplant recipients remains limited (7). The present study therefore evaluated digital access, perceived usefulness, trust, ethical concerns, and readiness for digitally supported follow-up in a real-world transplant cohort. We further explored whether responses differed according to sex, age, education, wearable use, and intensity of general technology use. The study was designed to identify implementation conditions that should be addressed prior to introducing data-driven or machine-learning-enabled precision-care pathways in this population.

## 2 Materials and methods

### 2.1 Study design and population

This cross-sectional observational study was conducted at the Heart Transplant Center of the Cardiac Surgery Unit, University Hospital of Padua, Italy. Consecutive adult heart transplant recipients attending routine outpatient follow-up during a 30-day recruitment period in April 2025 were invited to participate. Eligibility criteria were age 18 years or older and a history of heart transplantation. No exclusions were applied on the basis of time since transplantation, comorbidity, or digital competence, so as to reflect the heterogeneity of a real-world clinical population. Participation was voluntary and anonymous; no incentives were provided.

The study was approved by the local Ethics Committee (Approval No. 343n/AO/23) and conducted in accordance with the Declaration of Helsinki. Participants provided written informed consent prior to enrollment.

### 2.2 Questionnaire development and content

The questionnaire was developed specifically for this exploratory study rather and was not translated or formally adapted from a single validated instrument. Item generation was informed by themes consistently reported in the literature on digital health and telemedicine, technology acceptance and trust in healthcare, digital literacy, privacy, and data governance. The initial item set was tailored to the clinical context of heart transplantation by a multidisciplinary team including cardiac surgeons and a biomedical engineer.

The development process included expert review for relevance, clarity, and contextual appropriateness, followed by pilot testing in a small group of 5 heart transplant recipients. Pilot participants were asked to comment on item comprehensibility, wording, response options, and completion feasibility. Minor wording refinements were made before administration to the study cohort. This process supported face and content validity, but did not constitute formal psychometric validation.

The final instrument comprised sociodemographic items; questions on device access, general technology use, connectivity, and self-reported competence; and five-point Likert-scale items addressing perceived usefulness, safety, clinician communication and integration, medication-related confidence, preference for human advice, privacy, secondary data use, and consent control. The complete questionnaire is provided as Supplementary Material 1. As the survey intentionally spanned multiple conceptual domains and was not designed as a unidimensional scale, a single internal-consistency coefficient was not considered methodologically appropriate.

### 2.3 Statistical analysis

Categorical variables are reported as counts and percentages. Likert-scale responses were summarized across all five response categories; for prespecified subgroup analyses, scores of 4 or 5 were classified as agreement or strong agreement, and scores of 1–3 as non-agreement. Participants were grouped by sex, age (≤ 45, 46–55, and ≥ 56 years), education (lower secondary or less, high school, and university or postgraduate), wearable use, and daily general technology use (≤ 1, 2–5, and ≥ 6 h per day). Associations were assessed using Pearson chi-square tests or Fisher's exact tests when expected cell counts were small. Missing responses were excluded pairwise; denominators are reported where they differ from the total sample of 93. All tests were two-sided with a significance threshold of alpha = 0.05. As subgroup analyses were exploratory and multiple hypotheses were examined simultaneously, no multiplicity adjustment was applied; statistically significant findings should therefore be interpreted as hypothesis-generating rather than confirmatory.

## 3 Results

### 3.1 Participant characteristics and digital access

A total of 93 heart transplant recipients completed the survey (Table 1). The cohort included 61 men (65.6%) and 32 women (34.4%). Seventy-four participants (79.6%) were older than 45 years. Most had completed high school (55/93, 59.1%), 46 (49.5%) were employed, and 36 (38.7%) were retired. One participant did not report place of residence.

**Table 1:** Characteristics of heart transplant recipients enrolled in the study.

Characteristic	Category	*n* (%)
Sex	Male	61 (65.6)
	Female	32 (34.4)
Age	18–35 years	5 (5.4)
	36–45 years	5 (5.4)
	46–55 years	23 (24.7)
	56–65 years	26 (28.0)
	≥ 66 years	27 (29.0)
Education	Lower secondary or less	21 (22.6)
	High school	55 (59.1)
	University/postgraduate	16 (17.2)
	Other/not reported	1 (1.1)
Employment	Employed	46 (49.5)
	Retired	36 (38.7)
	Other	11 (11.8)
Digital access	Smartphone	85 (91.4)
	Personal computer	70 (75.3)
	Tablet	38 (40.9)
	Wearable	18 (19.4)

**Figure 1:**
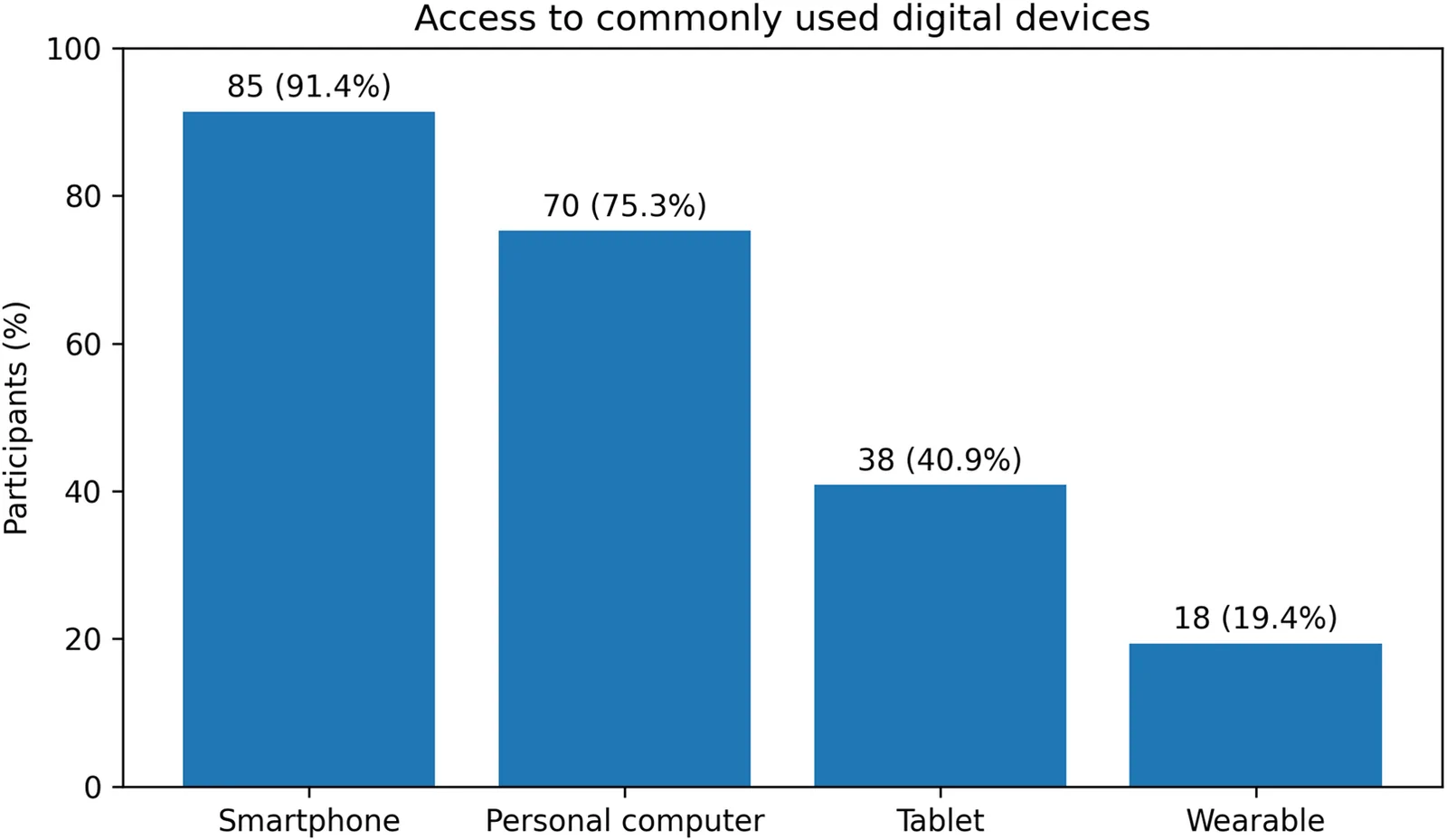
Access to commonly used digital devices among heart transplant recipients.

Digital access was widespread (Table 2 and Figure 1): 85 participants (91.4%) used a smartphone, 70 (75.3%) a personal computer, and 38 (40.9%) a tablet. Only 18 (19.4%) reported wearable-device use. Self-reported digital competence was beginner in 14 (15.1%), intermediate in 55 (59.1%), advanced in 20 (21.5%), and expert in four (4.3%). Among wearable users, none identified as a beginner, while 10/18 (55.6%) identified as advanced or expert.

**Table 2:** Selected exploratory subgroup comparisons. Percentages use available-case denominators. *P*-values are unadjusted and hypothesis-generating.

Comparison	Outcome	Agreement by subgroup	*p*-value
Sex	Technology improves monitoring	Female 19/32 (59.4%); male 50/60 (83.3%)	0.023
Age	Medication-related insecurity	<=45: 0/19; 46–55: 2/21 (9.5%); >=56: 13/53 (24.5%)	0.029
Age	Prefer physician advice	<=45: 5/19 (26.3%); 46–55: 13/21 (61.9%); >=56: 31/52 (59.6%)	0.030
Daily technology use	Medication-related insecurity	<=1 h: 5/16 (31.3%); 2–5 h: 10/47 (21.3%); >=6 h: 0/30	0.009
Daily technology use	Discomfort with device monitoring	<=1 h: 8/16 (50.0%); 2–5 h: 17/47 (36.2%); >=6 h: 4/30 (13.3%)	0.022
Daily technology use	Prefer physician advice	<=1 h: 10/16 (62.5%); 2–5 h: 29/46 (63.0%); >=6 h: 10/30 (33.3%)	0.029

### 3.2 Perceived usefulness, trust, and clinician integration

Three quarters of participants (69/92, 75%) agreed that technology could improve health monitoring, and 54/93 (58.1%) reported that health technology made them feel safer. Digital tools were widely viewed as useful for patients-clinicians communication (72/93, 77.4%). Clinician integration emerged as a central condition for trust: 69/93 (74.2%) agreed that an application or device would be more useful, effective, and reliable if it incorporated guidance from their treating clinicians.

**Figure 2:**
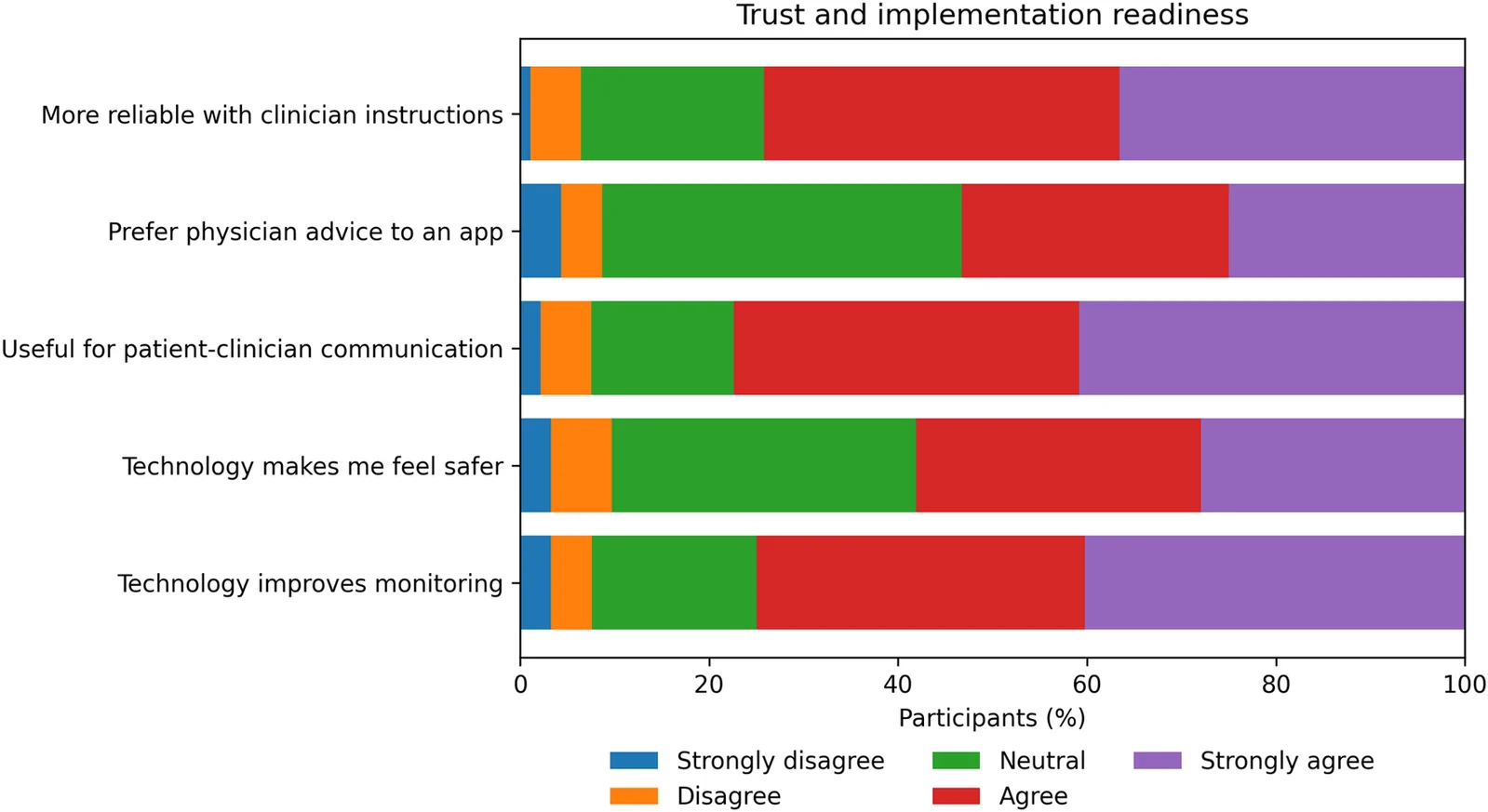
Distribution of Likert-scale responses for selected trust and implementation-readiness items.

Openness to digital health coexisted with a preference for human care. Twenty-nine participants (31.2%) expressed discomfort with device-led health monitoring in place of a physician oversight; 49/92 (53.3%) preferred receiving advice directly from a physician rather than relying on an application, and 64/93 (68.8%) preferred contacting their physician rather than relying on app-based notifications when concerned about their health. Medication management represented a distinct high-stakes domain: 15/93 (16.1%) agreed that they felt unsafe using technology to manage medications due to fear of errors (Figure 2).

### 3.3 Privacy and data governance

**Figure 3:**
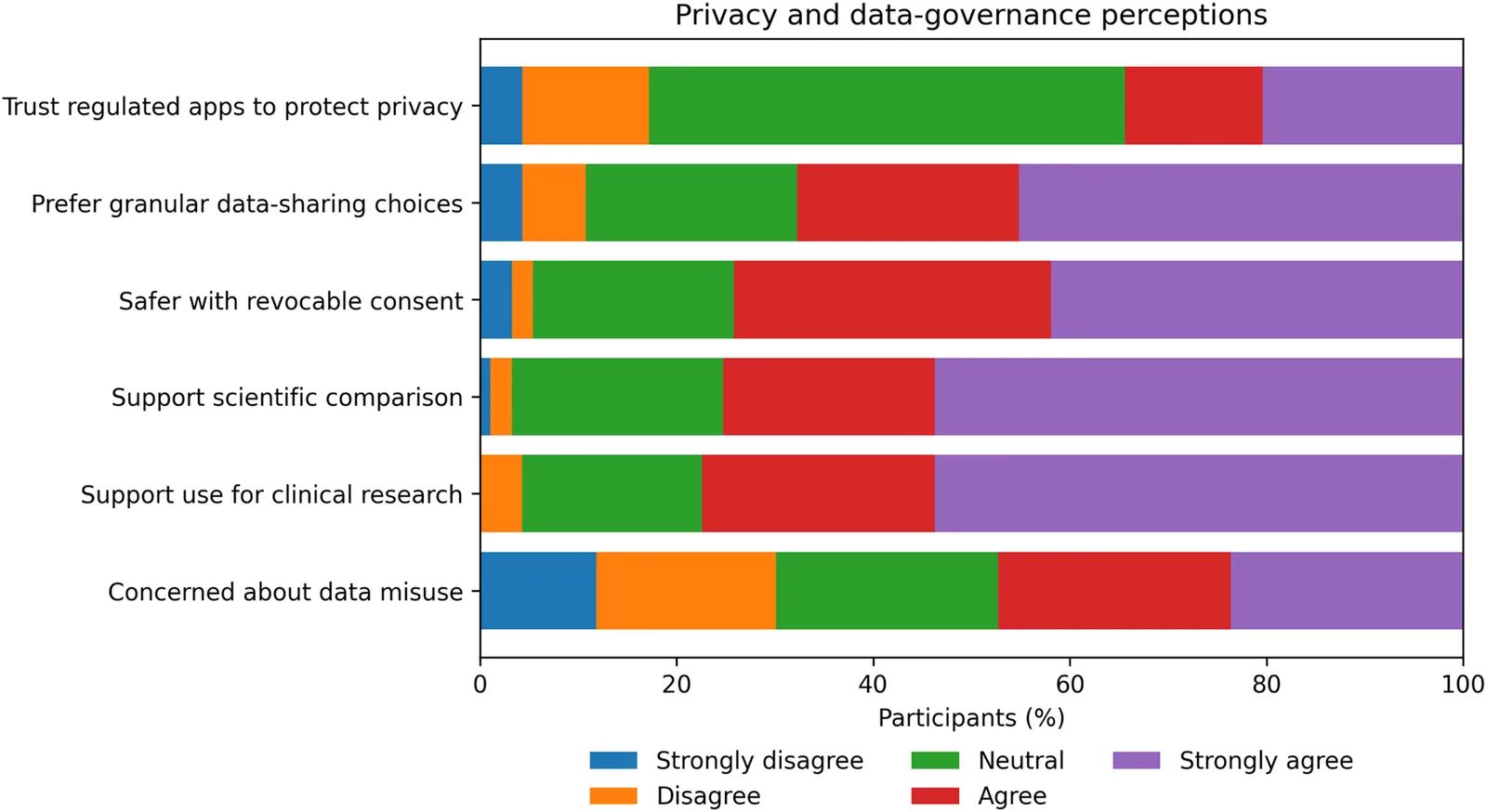
Distribution of Likert-scale responses for selected privacy and data-governance items.

Forty-four participants (47.3%) expressed concern that their data could be misused or shared without consent. Only 28/93 (30.1%) reported always reading privacy policies, and 32/93 (34.4%) trusted regulated applications and devices to protect personal information. In contrast, participants demonstrated substantial conditional willingness to contribute data: 72/93 (77.4%) supported the use of their data for clinical research, 70/93 (75.3%) supported comparison with other patient populations for scientific purposes, and 56/92 (60.9%) were more comfortable sharing data with healthcare professionals than with private companies. Patient-controlled governance mechanisms were strongly valued: 69/93 (74.2%) reported feeling safer when consent could be revoked at any time, and 63/93 (67.7%) preferred having the ability to choose which data to share and with whom (Figure 3).

### 3.4 Exploratory subgroup analyses

Men more frequently agreed that technology could improve health monitoring than women (50/60, 83.3% vs. 19/32, 59.4%; *p* = 0.023). No statistically significant sex differences were identified for the remaining prespecified trust and privacy outcomes.

Age was associated with medication-related insecurity (*p* = 0.029) and preference for physician advice over app-based guidance (*p* = 0.030). Agreement with medication-related insecurity was observed in 0 of 19 participants (0%) aged ≤ 45 years, 2 of 21 (9.5%) aged 46–55 years, and 13 of 53 (24.5%) aged ≥ 56 years. Preference for physician advice was reported by 5 of 19 (26.3%), 13 of 21 (61.9%), and 31 of 52 (59.6%), respectively.

**Figure 4:**
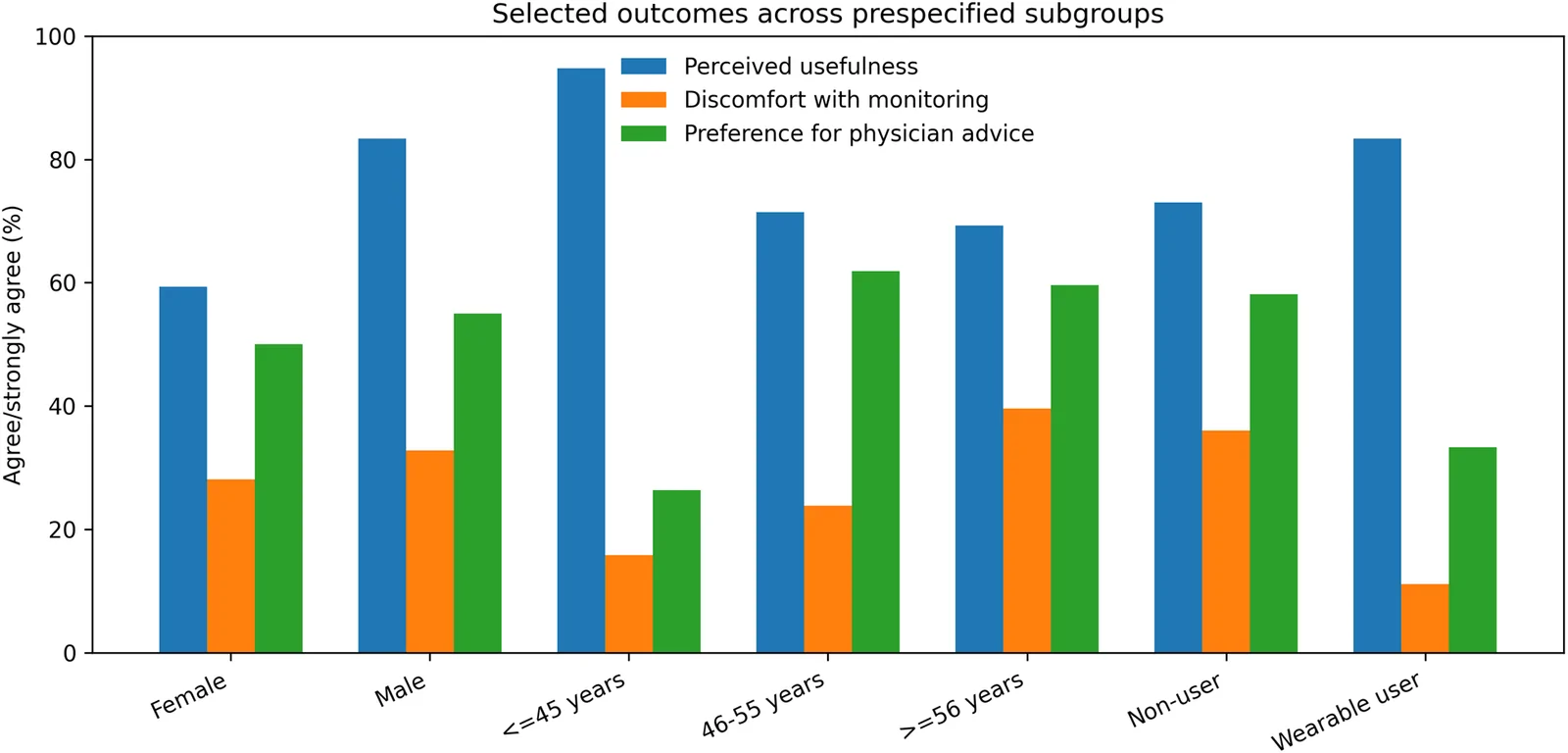
Selected outcomes across sex, age, and wearable-use subgroups.

Daily technology use intensity was associated with medication-related insecurity (*p* = 0.009), discomfort with device-led monitoring (*p* = 0.022), and preference for physician advice (*p* = 0.029). No participant using technology for ≥ 6 h per day reported medication-related insecurity, compared with 10 of 47 (21.3%) among those using it for 2–5 h per day and 5 of 16 (31.3%) among those using it for ≤ 1 h per day. Wearable use was not significantly associated with dichotomized trust outcomes; however, wearable users demonstrated a more advanced self-reported competence profile and lower medication-related insecurity in an exploratory ordinal comparison. Education was associated with trust that regulated applications adequately protect personal data (*p* = 0.004); however, sparse categories and the overall exploratory design warrant cautious interpretation of this finding (Table 2 and Figure 4).

## 4 Discussion

### 4.1 Principal findings

This real-world survey identifies a pattern of conditional readiness for digital health adoption following heart transplantation. Smartphone and computer access was high, and most recipients recognized the potential usefulness of digital monitoring. Nevertheless, wearable adoption remained limited, confidence varied according to the clinical task, and a substantial proportion of participants expressed concern about privacy or the prospect of technology displacing physician involvement. The predominant implementation signal was not unconditional enthusiasm for automation, but rather preference for tools embedded within clinician-led care and governed by transparent, patient-controlled, reversible data arrangements.

### 4.2 Trust is relational and task-specific

The findings suggest that trust should not be treated as a stable personal attitude toward technology. Participants could simultaneously endorse the usefulness of digital monitoring and express a preference for direct physician advice, particularly for medication management or the recognition of possible deterioration. This apparent ambivalence is clinically coherent: low-risk self-monitoring tasks differ from decisions that may carry immediate consequences for an immunosuppressed transplant recipient. Implementation strategies should therefore distinguish supportive functions, such as reminders and patient–clinician communication, and functions perceived as autonomous clinical decision-making.

The high value assigned to clinician-authored or clinician-endorsed content underscores the importance of relational integration in digital health design. Digital systems are more likely to gain acceptance when presented as extensions of the transplant team rather than replacements for it. This perspective supports the development of co-designed workflows in which alerts, recommendations, and escalation pathways are explicitly linked to known professionals and integrated within routine transplant services.

### 4.3 Digital experience, age, and equity

Greater daily use of technology was associated with lower medication-related insecurity and less discomfort with device-led monitoring. Age-related differences were most evident for high-stakes tasks and preference for professional advice, rather than for general statements about the future of technology. These observations argue against a simple young-versus-old digital divide. Instead, readiness appears to reflect experience, task complexity, perceived consequences, and access to support.

Wearable users represented a digitally self-selected subgroup: none identified as beginners and more than half described themselves as advanced or expert. Although dichotomized outcome differences were not statistically significant, this pattern highlights a potential implementation bias. Programs that rely on voluntary uptake may disproportionately enroll already confident users. Structured onboarding, assisted setup, training for patients and caregivers, and non-digital alternatives are therefore necessary to avoid widening disparities.

### 4.4 Data governance as an implementation condition

Participants were not uniformly opposed to data sharing. On the contrary, most supported the use of their data for clinical research and scientific comparison, provided that meaningful safeguards and control were in place. Fear of misuse coexisted with willingness to contribute data, indicating that the relevant question is not simply whether patients will share their data, but rather under what governance conditions they are prepared to do so. Revocable consent, granular choices, clear identification of data recipients, and separation of clinical and commercial uses should therefore be treated as core design requirements for any digital health implementation in this population.

### 4.5 Implications for precision care

**Figure 5:**
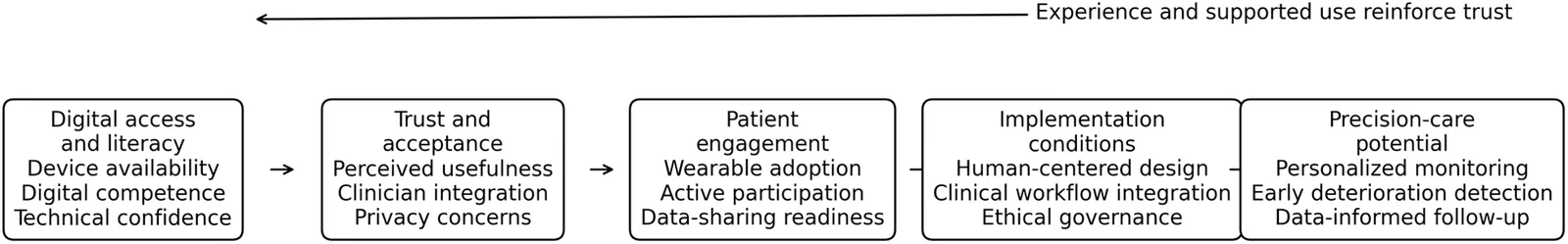
Conceptual framework linking digital access, trust, engagement, implementation conditions, and the potential for precision care.

Longitudinal patient-generated data may ultimately support personalized surveillance and the earlier identification of clinically relevant change. However, algorithmic performance alone will not ensure adoption. The conceptual pathway proposed in Figure 5 positions digital access and literacy as upstream determinants of trust, engagement, and implementation readiness. Only when these elements are aligned can patient-generated data contribute reliably to precision-care pathways in transplant medicine. This framework is conceptual in nature and requires prospective evaluation; nonetheless, it translates the present survey findings into practical implementation domains that can inform the design of future digital health programs.

### 4.6 Strengths and limitations

Strengths of this study include its focus on an underrepresented, clinically complex population, consecutive recruitment in routine follow-up, comprehensive assessment of technological, relational, and ethical dimensions, and a multidisciplinary, literature-informed questionnaire development process incorporating expert review and patient pilot testing.

Several limitations should be acknowledged. The study was conducted at a single center and the relatively small sample size limited precision and subgroup statistical power. Responses were self-reported and may be affected by selection, recall, or social-desirability bias. The cross-sectional design precludes causal inference, and self-reported attitudes may not reliably predict sustained use. The questionnaire underwent face and content review but did not undergo formal psychometric validation; future studies should evaluate reproducibility, construct validity, and responsiveness. Multiple exploratory comparisons were performed without adjustment, increasing the probability of chance findings. Finally, potentially relevant clinical variables such as time since transplantation, comorbidity, prior telemonitoring exposure, and caregiver support, were not incorporated into the present analysis and should be addressed in future work.

## 5 Conclusions

Heart transplant recipients demonstrate substantial but conditional readiness for digitally supported follow-up care. High device access and perceived usefulness do not automatically translate into adoption. Clinician integration, task-specific confidence, and transparent, patient-controlled data governance emerge as central determinants to successful implementation. Future programs should adopt a staged introduction approach, incorporate supported onboarding, establish clear escalation pathways, and include prospective evaluation of both clinical outcomes and equity. Larger, multicenter studies are needed to confirm these findings and to inform the design of scalable, human-centered digital health pathways in heart transplantation.

## Data Availability

The original contributions presented in the study are included in the article/Supplementary Material, further inquiries can be directed to the corresponding author.
