# Supplementary material for: Patient trust and readiness for digital health-enabled precision care after heart transplantation: a real-world survey
Source: Front Digit Health. 2026 Aug 11;8:1839950. doi: 10.3389/fdgth.2026.1839950 (PMC13503613; doi:10.3389/fdgth.2026.1839950)
Supplement: Supplementary file 1 [file Datasheet1.docx]

# Supplementary Material 1 Digital Health Trust and Readiness Questionnaire

## Section 1. Demographic Characteristics

- 1.1 Sex: Male; Female; Non-binary; Prefer not to answer
- 1.2 Age: 18–25; 26–35; 36–45; 46–55; 56–65; ≥66; Prefer not to answer
- 1.3 Educational Level: Primary education; Lower secondary; Upper secondary; Bachelor’s degree; Master’s degree; Postgraduate degree
- 1.4 Employment Status: Employed; Student; Retired; Unemployed; Other; Prefer not to answer
- 1.5 Marital Status: Single; Married; Divorced; Widowed; Prefer not to answer
- 1.6 Place of Residence: >100,000; 50,000–100,000; 20,000–50,000; 5,000–20,000; <5,000; Prefer not to answer

## Section 2. Access to and Use of Technology

- 2.1 Level of technology use: Beginner; Intermediate; Advanced; Expert
- 2.2 Devices used daily (Yes/No/Not sure): Desktop; Laptop; Smartphone; Tablet; Wearable devices
- 2.3 Internet connection: Excellent; Good; Moderate; Poor; No access
- 2.4 Confidence in solving technological problems: Very confident; Fairly confident; Not very confident; Not at all confident
- 2.5 Average daily hours of technology use: Open numeric response (0–24)
- 2.6 Primary use of technology: Only work; Mostly work; Equal work/leisure; Mostly leisure; Only leisure

## Section 3. Digital Health Technologies

- 3.1 Use of health-related digital tools: Several frequently; Several occasionally; Few frequently; Few rarely; Not using but interested; Not using and not interested
- Likert scale (Strongly disagree – Disagree – Neutral – Agree – Strongly agree):
- Technology helps me monitor my health better.
- Using technology for health makes me feel safer.
- I am afraid of making mistakes managing medications with technology.
- I feel uncomfortable if a device monitors my health instead of a doctor.
- Digital tools are useful for communication with my doctor.
- I prefer physician guidance over app-based advice.
- I would feel uncomfortable if an app collected emotional data.
- Health technologies represent the future of medicine.
- I prefer calling my doctor rather than relying on app notifications.
- App instructions are harder to understand than direct medical advice.
- Notifications help me reach recommended health goals.
- Health apps are more useful for younger people.
- I do not trust my ability to use health apps effectively.
- I prefer paper over electronic recording of medical data.
- Apps including physician instructions would be more reliable.

## Section 4. Data Collection and Privacy (Likert scale)

- I consider privacy policies when using health apps.
- I read privacy policies before downloading apps.
- Health data are used only to improve my health.
- I am unsure how my data are used and this concerns me.
- I agree data may be used for clinical research.
- I agree data may be compared with other individuals’ data.
- I agree data may be shared with third parties.
- I fear my data may be misused.
- I feel safer if I can withdraw consent at any time.
- I am not comfortable sharing health data with apps.
- I am comfortable sharing data with healthcare professionals but not private companies.
- I prefer choosing which data to share and with whom.
- Regulated apps adequately protect my privacy.
